# Evaluation of upper labial frenectomy: A randomized, controlled comparative study of conventional scalpel technique and Er:YAG laser technique

**DOI:** 10.1002/cre2.374

**Published:** 2020-12-25

**Authors:** Roxana Sarmadi, Pia Gabre, Andreas Thor

**Affiliations:** ^1^ Department of Paediatric Dentistry Public Dental Health, Uppsala County Council Uppsala Sweden; ^2^ Department of Cariology Institute of Odontology, The Sahlgrenska Academy of Gothenburg Gothenburg Sweden; ^3^ Department of Preventive Dentistry Public Dental Health, Uppsala County Council Uppsala Sweden; ^4^ Department of Plastic and Oral and Maxillofacial Surgery, Department of Surgical Sciences Uppsala University Uppsala Sweden

**Keywords:** Er:YAG laser, labial frenectomy, patients' experiences, wound healing

## Abstract

**Objectives:**

Abnormalities in the maxillary frenum may lead to esthetic or functional limitations and need to be corrected with a surgical intervention called frenectomy. The aim of the study was to compare frenectomies performed using Er:YAG laser technology with those using a conventional scalpel technique. Comparisons were of patients' experiences, treatment times, bleeding during treatment and wound healing.

**Material and methods:**

The trial was performed as a prospective, randomized and controlled, single‐blind investigation. A total of 40 patients requiring frenectomy were randomly assigned to groups which underwent either conventional or Er:YAG laser treatment. Patients' experiences, treatment time, bleeding and wound healing were evaluated immediately after surgery and 5 days, 12 days and 3 months after surgery.

**Results:**

Significant increase in time spent in surgery and bleeding was seen with conventional scalpel surgery. Directly after surgery the wound area was significantly larger in the laser group but at the 5‐day evaluation no difference could be observed between the groups. Finally, patients were satisfied with both methods, giving them the same assessments.

**Conclusion:**

In the frenectomy procedure, laser surgery is faster and causes less bleeding and may be advantageous in frenectomies.

## 1 INTRODUCTION

The maxillary midline frenum is an anatomical structure that connects the mucosa of the alveolar process to the upper lip (AR, TH, & Carranza, 2002). The frenum may limit the mobility of the lip, affecting chewing and speech. In addition, the frenum is sometimes too closely attached to marginal gingiva and inhibits optimal brushing. Thus, under unfavorable conditions, there is a future risk of gingival retraction due either to plaque accumulation or muscle pull (Delli, Livas, Sculean, Katsaros, & Bornstein, 2013). In 1971, Sewerin classified maxillary midline frenum into eight categories (Sewerin, 1971), but in 1974 Mirko et al. presented a new classification system divided into four categories based on the interaction between the periodontal tissue and frenum (Mirko, Miroslav, & Lubor, 1974). An abnormal frenum is closely associated with a number of syndromes and may have a role in the development of midline diastema (Delli et al., 2013), a condition that can be regarded differently from an esthetic point of view according to cultural background (Akinboboye, Umesi, & Ajayi, 2015). Some authors regard variations in the maxillary labial frenum as inherent, and do not see it as a pathological condition (Townsend, Brannon, Cheramie, & Hagan, 2013).

Histologically the frenum consists of collagen tissue, elastic fibres and often muscle fibres (Ross, Brown, & Houston, 1990). The aberrant frenum can be treated by a surgical procedure called frenectomy which means complete removal of the frenum, including its attachment to the underlying periosteum. This can be carried out using a conventional scalpel technique, electro surgery or by lasers. The classical frenectomy is described as an excision including interdental tissues and the palatine papilla, to ensure that muscle fibres are removed. Modifications to the classic frenectomy were developed, such as Miller's technique used for post‐orthodontic diastema cases, Z‐plasty procedure used when the frenum has a low insertion combined with a short vestibulum, and V‐Y plasty which is used for lengthening the area (Devishree & Shubhashini, 2012).

Laser technology has been used in oral surgery since the early 1990's and several authors have reported its use, using different wavelengths, for frenectomies (Kravitz & Kusnoto, 2008; Olivi et al., 2011; Pick & Colvard, 1993; Pie‐Sanchez, Espana‐Tost, Arnabat‐Dominguez, & Gay‐Escoda, 2012). According to previous studies, patients who undergo a laser frenectomy have less post‐operative discomfort than patients treated by conventional procedures (Cervetto, Villar, & Ellis, 2011; Haytac & Ozcelik, 2006; Uraz, Çetiner, Cula, Guler, & Oztoprak, 2018). In a 3 year follow‐up of 156 children who had a frenectomy performed with Er,Cr:YSGG laser, 8% had recurrences that required interventions. However, the conclusions were that the method was safe and effective with no post‐operative side effects and with high patient acceptance (Olivi, Chaumanet, Genovese, Beneduce, & Andreana, 2010). A comparative study of Er, Cr:YSGG laser and CO_2_ laser has shown faster healing for a frenectomy performed with Er, Cr:YSGG laser, but the CO_2_ laser treatment resulted in a shorter operation and less bleeding during surgery (Pie‐Sanchez et al., 2012).

The aim of this study was to compare the frenectomy procedure when performed with Er:YAG laser technology and with conventional scalpel technique, taking into account wound healing, patients' experiences, treatment time and bleeding during treatment. The hypothesis was that a frenectomy using Er:YAG laser technology would lead to faster healing and, in addition, less bleeding and greater patient comfort.

## 2 MATERIAL AND METHODS

The study was approved by the Ethics Committee at the Faculty of Medicine, Uppsala University (No. 2014/253). It has also been registered in Clinicaltrials.gov (NCT03104764). All participants and their legal guardians received verbal and written information about the study and informed consent was obtained from all guardians before the study started.

### 2.1 Study design

The trial was performed as a prospective, randomized and controlled, single‐blind investigation to study clinical parameters and patients' experiences when performing a frenectomy, comparing Er:YAG laser treatment with conventional scalpel technique.

### 2.2 Participants

All patients in the Swedish public dental service, between 7 and 19 years of age, and who were referred to the specialist paediatric dentistry clinic in Uppsala for frenectomy were asked to participate in the study. For all patients under the age of 18, legal guardians were also asked for permission.

Patients and legal guardians confirmed participation in the study by signing the informed consent form. Inclusion criteria for participation were: (a) patients between 7 and 19 years old, (b) patients were referred to the specialist clinic for paediatric dentistry, and (c) patients were in need of frenectomy for the upper labial frenum.

The researcher responsible for the study (RS), an experienced dentist specializing in paediatric dentistry, examined all patients and took the final decision whether the patient met the inclusion criteria or not. Exclusion criteria in the study were: (a) patients with severe general diseases (ASA >2; Doyle & Garmon, 2017), (b) patients who required general anesthesia during treatment and (c) patients who smoked.

### 2.3 Randomization procedure

A total of 40 envelopes, divided into four blocks containing ten envelopes each, were prepared for the randomization procedure. One of two alternative texts was placed in each envelope in order to randomly allocate the patients to the conventional scalpel group or the Er:YAG laser group.

### 2.4 Pre‐treatment procedure

All patients were called for a clinical examination, review and detailed information about the planned procedure. A health history was taken from parents and patients before surgery. If needed, apical radiographs were made to exclude supernumerary teeth and odontogenic tumors. The distance between the insertion of the frenum and the highest point of papilla was measured using a caliper. The area was photographed using a standard photography technique prior to the surgery. A system camera (Nikon AF‐S DX Micro‐Nikkor 85 mm f/3.5G ED VR, Japan) was used with preset manual settings: 60 mm lens, scale 1:1, distance 0.4 m, mode F32. The image was taken at the distance for optimum sharpness. When photographs were taken, the same settings were used for all patients on all occasions. To verify that photographs were standardized, the incisal edge of tooth 11 was measured with a caliper in all patients.

### 2.5 Treatment procedure

The main researcher (RS) performed all surgical treatments. All patients received 0.9 ml local infiltration anesthesia (Xylocain Dental Adrenalin 20 mg/ml + 12,5 microg/ml, Dentsply, London, UK) which was administered with a computer assisted injection system (the Wand, Mildstone Scientific, Livingstone, NJ).

When laser technique was used, Er:YAG laser, with the wavelength 2,940 nm, (AT Fidelis plus 3, Fotona, Slovenia) with handpiece R014 and cylindrical, sapphire fiber tip 8/1.3 mm was used. The settings were, pulse duration VLP mode (1,000 μs), pulse energy 150 mj, pulse frequency 10 Hz without supply of air and water. The settings were in accordance with the manufacturer's recommendations for use in frenectomy and the clinician's (RS) clinical experience. Protective glasses covering wavelength 2,940 nm were worn both by the professionals and the patient. A sterile disposable scalpel with blade no. 15 (stainless steel blade with safety cap, Aditiya Dispomed Products Pvt. Ltd. Made in India) and absorbable suture 4.0 (Johnson & Johnson, New Brunswick, NJ) were used in all cases in the conventional scalpel group. The same absorbable suture was used in only two cases in the Er:YAG laser group, to stop bleeding. The duration of surgery was measured with a stopwatch from the time the therapist initiated the procedure with laser or scalpel until the surgery was complete, including suturing and hemostasis. The same surgical technique was used for both methods. An incision following the vertical axis of the frenum was made and then an angled cut, so that the wound took a rhomboidal shape. Excessive mucosa inside the upper lip was removed. If the frenum attached very low between central incisors and had contact with the papilla incisiva, this part was also removed.

Bleeding during surgery was measured by letting sterile compresses absorb the blood. Before surgery five compresses were weighed in a stainless‐steel vessel on a balance capable of measuring with an accuracy of hundredths of a gram (TL 402 Toploading Balances, Denver Instrument Companies, CO). As the compresses were used, they were put back into the vessel. After completion of the treatment, the difference in weight before and after surgery was noted.

### 2.6 Post‐treatment procedure

Immediately after the surgery the patients were asked to answer questions about the treatment. In a questionnaire, patients were asked for their views regarding visiting a dentist, their feelings about receiving local anesthesia, and their experiences of the completed treatment. Participants were asked to consider four statements and to mark disagreement/agreement in a visual analogue‐scale (VAS). In some cases, parents helped patients complete the survey, but in all cases the patient answered the questions and marked the VAS scale.

The patients received written information about how to perform postoperative oral care. The wound was to be gently bathed with a chlorhexidine solution (Hexident 1 mg/ml, Meda AB, Solna, Sweden) twice a day for 10 days. During this period the teeth were to be brushed with a toothpaste without the agent sodium lauryl sulfate (Zendium, Unilever Sverige AB, Solna, Sweden).

### 2.7 Evaluation

The patients returned for evaluation 5 days, 12 days and 3 months after surgery. The 5 and 12‐day post‐surgery evaluations were done by the dentist who had performed the operation (RS). After 5 days, the sutures were removed. The evaluation 3 months after surgery was performed by a specialist in oral and maxillofacial surgery (AT), who was not informed of which surgical technique had been used. On the follow‐up occasions the patients answered a questionnaire on their opinion of the treatment, any symptoms after treatment, and if they had needed analgesics or antibiotics for postoperative complications. Standardized photographs of the treated area were also taken on the follow‐up occasions—5 and 12 days, and 3 months after surgery.

Wound healing was evaluated by measuring the size of the surface that was not covered by epithelium on the standardized photographs. The photos were exported from the patient journal to a file in JPEG format, converted into PDF format and were then transferred to photo editing software (Adobe Acrobat Reader DC, Adobe Systems Software, Ireland, Ltd.). Using the software, the specialist paediatric dentist (RS) marked the surface that was not covered with epithelium and then the program calculated the size of the surface. The real size of wound surface in square mm was calculated by using the actual length of the incisal edge of tooth 11. Photos belonging to ten patients were selected and the measurements of wound area were repeated three weeks after the first measurement. The first and second measurements of the wound area were used to calculate intra‐examiner reliability.

**FIGURE 1:**
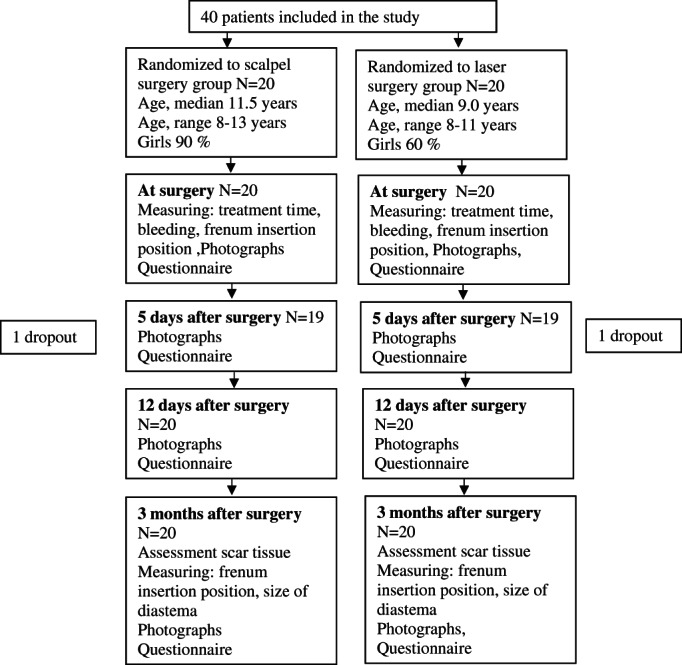
Flow‐chart of the study including the age and gender of participants

All data before, during and after the treatment was gathered in a protocol. A flowchart shows the different parts of the study, the number of participants and the dropouts (Figure 1).

**FIGURE 2:**
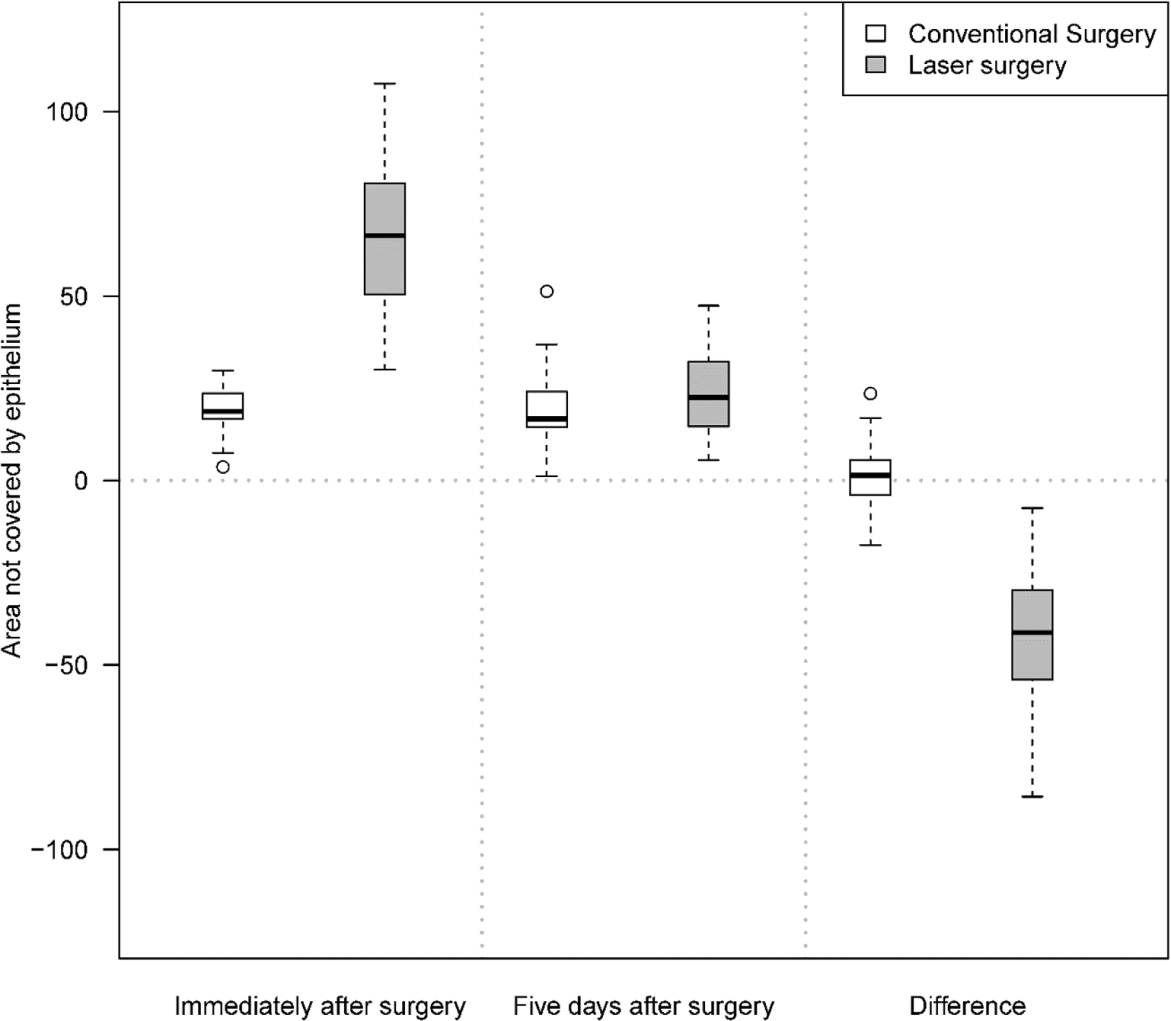
The area not covered by epithelium immediately after surgery and 5 days after surgery, in conventional surgery group and laser surgery group (in square millimeters)

### 2.8 Statistical analysis

A power calculation was made to determine the number of participants in the study. The calculation was based on the primary outcome, wound healing, measuring the time it would take for the wound to be covered by epithelium. We estimated that the coverage would take place 3 days faster after laser surgery. A sample size of 20 in each group was calculated to have 80% power with a significance level of .05 to detect this difference. Thus, a total of 40 patients was included in the study.

Baseline characteristics were presented with means or medians for continuous variables and percentages for categorical. Statistical comparisons between baseline variables were performed using t‐tests and Chi‐Squared tests where appropriate. Patient perception VAS answers, amount of blood, difference between groups and wound areas were compared using t‐tests. Analyses of changes in the distance between frenum attachment and diastema were performed using linear regression, with the 3‐month value as a dependent variable while adjusting for the baseline (0 days) value. In order to ensure that the examiners' measurements of the area that was not covered by epithelium on the standardized photographs was recorded in a reliable way (intra‐examiner reliability), intra‐class correlation was used. Statistical analyses were performed using R v3.5.0 (R Foundation for Statistical Computing, Vienna, Austria).

## 3 RESULTS

A total of 53 patients were asked if they wanted to participate in the study, out of which nine chose not to participate indicating lack of time and long journeys as reasons. Three patients were excluded at the first examination because there was insufficient indication for a frenectomy, and one patient was excluded due to being a smoker. A total of 40 patients met the inclusion criteria and were consecutively included in the study. After randomization, participants were allocated to groups for conventional surgery and laser surgery, with 20 patients in each group. Figure 1 shows the gender and age of participants, as well as participation in the different parts of the study, in a flow diagram. There was a larger proportion of girls in the conventional surgery group than the laser group (*p* < .028 Chi‐Squared test). One patient in each group took pain relief drugs prior to the surgery. Directly after treatment patients marked on a VAS scale in the questionnaire how uncomfortable it was to go to the dentist and receive local anesthesia in general (0 not uncomfortable and 100 very uncomfortable). In general, patients did not feel uncomfortable meeting a dentist (mean 18.0 and 17.2 for conventional scalpel and laser group, respectively. *p* = .914 for difference between groups). The patients reported higher discomfort when receiving local anesthesia, but overall the estimates were low (mean 26.3 and 33.5 for conventional scalpel and laser group, respectively. *p* = .469 for difference between groups).

**FIGURE 3:**
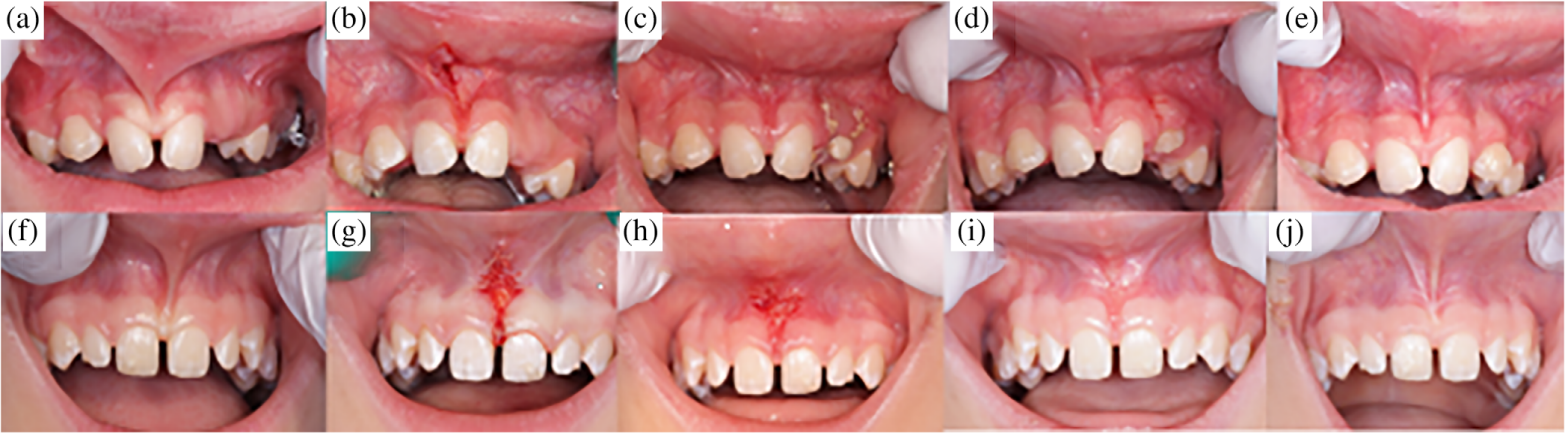
Clinical images showing labial frenectomy with Er:YAG laser technology (a–e) and with the conventional technique (f–j). a + f = before surgery, b + g = immediately after surgery, g + h = after 5 days, d + i = after 12 days, e + j = after 3 months

The distance between the frenum attachment and the highest point of the papilla varied between 0 and 3.5 mm before treatment and the size of midline diastema varied between 1.4 and 3.4 mm (Table 1). In the follow‐up 3 months later, the distance between the frenum attachment and the highest point of papilla was clearly longer, but there was no significant difference between the two groups when it came to measurements taken when the operation was performed and at the 3‐month control (Table 1). The mean of the midline diastema decreased by 0.97 mm in the conventional scalpel group and 0.62 mm in the laser group, a non‐significant statistical difference (Table 1). The mean duration of conventional scalpel surgery was 10 min 35 s and the mean duration of laser surgery was 6 min 52 s, which means conventional surgery was 54% longer (*p* < .001, Table 1). Bleeding during surgery was approximately three times as high in conventional scalpel surgery. However, bleeding was limited for both conventional and laser surgery (mean 1,080 mg and 332 mg respectively, *p* = .040, Table 1) In the conventional scalpel surgery group all patients needed suturing while in the laser surgery group only two of the 20 patients needed suturing. In all cases, except for one patient in the laser group, scar tissue could be seen after 3 months. No differences in scar tissue formation could be seen between the two techniques.

**TABLE 1:** Duration of the surgery, bleeding during the surgery, distance between frenum attachment and the highest point of the papilla, size of the midline diastema at surgery (Day 0) and after three months

Variable	Conventional surgery mean	Laser surgery mean	Laser vs. conventional estimate (95% CI)^a^	*p*‐Value
Duration of surgery (s)	635.0	412.0	(−295.51–150.18)	<.001
Bleeding during surgery (mg)	1,080	332	(−1,428.12–57.87)	.040
Distance frenum attachment—papilla (mm)				
0 days	1.92	1.71		
3 months	6.06	5.33		
Difference	4.14	3.62	−0.67 (−1.41–0.06)*	.081
Diastema (mm)				
0 days	2.43	2.33		
3 months	1.47	1.72		
Difference	−0.97	−0.62	0.31 (−0.07–0.70)*	.118

Abbreviation: CI, confidence interval.

^a^ Analyses were performed using *t*‐tests except for analyses marked with *, which were performed using linear regression with dependent variable value at 3 months, adjusting for the baseline (0 days) value.

Immediately after surgery, participants answered questions about discomfort and pain during the treatment, and whether they were satisfied with the treatment. On a VAS scale the estimates about discomfort and pain were low—the mean values were 13.0 and 12.5 on a scale 0–100 (0 not uncomfortable and 100 very uncomfortable, Table 2). Overall, the patients were satisfied with the treatment (mean 86.2 and 86.9 respectively, Table 2). Five days after the treatment 11 patients stated that they had taken pain relief drugs after conventional scalpel surgery and 9 patients after laser surgery. Twelve days after treatment the corresponding numbers were 10 and 8 patients, respectively. On the same occasion one patient stated that antibiotics were used after conventional scalpel surgery while none of the laser surgery patients had used antibiotics. During the period from 12 days to 3 months after surgery, no participants had used antibiotics. After 5 and 12 days, and 3 months, questions about how the treatment was experienced, how much pain occurred after treatment and how satisfied the patient was with the treatment gave equivalent estimates on the VAS scale for both groups, (Table 2).

**TABLE 2:** Participants' answers to questions about treatment immediately after, 5 days, 12 days and 3 months after surgery by marking on a VAS scale (0 not uncomfortable and 100 very uncomfortable)

	Conventional surgery	Laser surgery	Laser vs.conventional surgery	
Variable	mean	mean	estimate and 95% CI	*p*‐Value
Immediately after surgery				
Degree of discomfort during the treatment	13.05	12.55	−0.5 (−10.8 2–9.82)	.925
Degree of satisfaction with the treatment	86.2	86.9	0.8 (−10.7–12.2)	.899
5 days after surgery				
Degree of discomfort during the treatment:	18.0	21.2	3.2 (−12.253–18.677)	.686
Degree of pain during the treatment	27.2	22.4	−4.8 (−16.8–7.12)	.433
Degree of satisfaction with the treatment	92.16	86.3	−5.8 (−11.80–0.2223)	.067
12 days after surgery				
Degree of discomfort during the treatment	15.7	18.9	3.3 (−9.9 3–16.43)	.632
Degree of pain during the treatment	17.6	12.6	−4.9 (−16.2 3–6.43)	.402
Degree of satisfication with the treatment	93.2	89.2	−4.0 (−8.697–0.697)	.103
3 months after surgery				
Degree of discomfort during the treatment	16.2	22.1	6.0 (−8.0 2–20.00)	.407
Degree of satisfication with the treatment	83.5	86.3	2.9 (−89.980–14.687)	.640

*Note*: All analyses were performed using a *t*‐test.

Abbreviations: CI, confidence interval; VAS, visual analogue‐scale.

Immediately after surgery the wound area was clearly larger after laser surgery compared to conventional surgery (mean 66.8 and 19.1 square millimeters respectively, difference laser vs. conventional 47.7 mm, 95% CI 37.9–57.6, *p* < .001). The measurement of the area that was not covered by epithelium on the standardized photographs after 5 days showed no statistical differences between the groups (difference laser vs. conventional 4.1 mm, 95% CI −3.2 to 11.4, *p* = .280). After 12 days all wounds were covered by epithelium in both groups (Figures 2 and 3).

The test of intra‐examiner reliability showed a strong level of agreement. (In ten patients, post‐op intra‐class correlation (ICC) = 0.99 (95% CI 0.98–1.00) and at 5 days ICC = 0.89 (95% CI 0.65–0.97).

## 4 DISCUSSION

This study compared conventional scalpel surgery with laser surgery in performing a frenectomy of the upper labial frenum. Laser surgery took less time and resulted in less bleeding. On the other hand, we found no major differences between the groups at the 5‐day evaluation in the area not covered by epithelium, and there were no differences in the patients' perceptions of the two methods.

The study had a randomized, prospective, controlled and single‐blind design. In a single‐blind trial, only the examiner or only the patient is blind to the allocation and in this study the dentist who performed the 3‐month evaluation did not know which treatment group the individual belonged to. In the evaluation 5 and 12 days after surgery it was not possible to use a blinded examiner since it was obvious due to the appearance of the wound which method was used. It would have been desirable that the patient was also unaware of the method used, but it was impossible to conceal this from the patient. In the first questionnaire, answered immediately after treatment, the participants estimated the discomfort associated with visiting a dentist in general, and with receiving local anesthesia. There is a risk that patients' answers were influenced by their recently completed treatment instead of being an estimate of their total experience as dentistry patients. Swedish children are often caries‐free and have rarely experienced more advanced treatment, such as local anesthesia. Thus, the answers may have been more valid if they had been given before treatment. Another uncertainty is that it is difficult to assess to what extent the parents affected the answers, especially for the youngest children. The participants in the conventional scalpel group tended to be older than the laser group, although this was not a statistically significant difference. However, both groups evaluated the treatment methods very similarly. We had one dropout due to illness in each group in the 5‐day follow‐up, but all patients participated in the 12‐day and 3‐month follow‐ups and this is a strength of this study.

Patients were generally satisfied with the surgical treatment, regardless of method and did not experience it as unpleasant or particularly painful. This result differs from other studies where patients experienced less discomfort after frenectomy with laser surgery (Cervetto et al., 2011; Haytac & Ozcelik, 2006; Uraz et al., 2018). In a review of laser surgery of soft tissue in orthodontics eight studies were included, of which four described frenectomy and four gingivectomy, decreased post‐operative pain was reported after laser surgery (Seifi & Matini, 2017). A similar result are reported in a recently published systematic review where a meta‐analysis of six studies showed less pain 1 day after frenectomy performed by laser compared to conventional scalpel technique while there were no differences 7 days after treatment (Protásio, Galvão, & Falci, 2019). The result also differs from studies comparing excavation of caries tissue compared with rotary bur since patients, and especially children, prefer the use of the laser to the rotary bur (Keller et al., 1998; Sarmadi, Hedman, & Gabre, 2014). In the present study, there were no differences in pain reported immediately after treatment and on all follow‐up occasions. Pain levels were slightly higher 5 days after surgery than 12 days after. The reported use of analgesics supports the answers in the questionnaire, that is, the pain experience was higher in the 5 days following surgery and there were no differences between the two methods.

Laser surgery was significantly shorter, and surgery executed with a conventional scalpel took 54% longer. The mean duration of conventional scalpel surgery in this study was 10 min 35 s and the mean duration of laser surgery was 6 min 52 s. Previous studies have reported time differences between different surgical techniques when soft oral tissue is removed. Pie‐Sanchez et al. (2012) found that surgery took approximately three times longer when a frenectomy was performed with an Er, Cr:YSGG laser than a CO_2_ laser and Medeiros Junior, Gueiros, Silva, de Albuquerque Carvalho, and Leao (2015) reported a surgery time of 30% longer when scalpel surgery was used compared to Nd:YAG laser. On the other hand, several studies have reported no time differences between different surgical procedures in soft tissue surgery. Ize‐Iyamu, Saheeb, and Edetanlen (2013) compared conventional scalpel surgery with diode laser and Suter, Altermatt, and Bornstein (2017) compared CO_2_ laser with Er:YAG laser, and neither study reported time differences. In the recently published review by Protásio et al. (2019) a meta‐analysis showed significantly shorter surgery time for laser compared to scalpel technique. Considering that the time difference between the two methods in our study was statistically significant with approximately 4 min, this is a great advantage for the laser method as shorter surgery duration in this age group greatly facilitates treatment for the patient and the therapist as well.

Although both techniques resulted in limited bleeding, the scalpel surgery resulted in approximately three times as much bleeding as the laser method. Bleeding in soft tissue surgery has been reported in several studies but the method of measuring bleeding has varied, such as different rating scales of four or five levels to assess the bleeding. This differs from our study where bleeding was measured more exactly, by weighing the blood. Several studies have compared conventional scalpel technique with different types of lasers and all report significantly less bleeding when laser is used (Ize‐Iyamu et al., 2013; Medeiros Junior et al., 2015; Olivi, Genovese, & Olivi, 2018; Sobouti, Rakhshan, Chiniforush, & Khatami, 2014). Two further studies, when comparing different types of lasers, have reported bleeding after various soft tissue surgical procedures (Pie‐Sanchez et al., 2012; Suter et al., 2017). Suter et al. noted that intraoperative bleeding was more common after Er:YAG surgery than after CO_2_ laser (Suter et al., 2017). Although the main indication for Er:YAG laser is removal of hard tissue it can also be used for soft tissue surgery. In this study Er:YAG laser was used since this type of laser was available in Public Dental Health in Uppsala county.

Follow up 3 months after surgery, scar tissue formation was registered in all patients except one in the laser group. A review including eight studies concludes that no significant differences between conventional scalpel surgical technique and surgical laser could be seen in treatment outcome (Seifi & Matini, 2017). The midline diastema had decreased by 0.97 mm in the scalpel surgery group and by 0.62 mm in the laser group without showing any statistically significant differences. Four patients in the scalpel surgery group started active orthodontic treatment before the 3‐month evaluation. This may explain the reason why midline diastema decreased more in the conventional scalpel group. Ize‐Iyamu et al. showed that all midline diastema had closed several years after frenectomy, but when frenectomy and orthodontic treatment were combined, the closing process was faster (Ize‐Iyamu et al., 2013). In their follow‐up between 4 and 19 months after surgical treatment, 35% of the patients treated with both surgery and orthodontic treatment had a persistant diastema, while 89% of those treated only with surgery had a diastema that persisted (Ize‐Iyamu et al., 2013). In the present study, the distance between frenum insertion and the highest point of papilla had increased significantly in the evaluation carried out 3 months after surgery, and the increase in both groups was similar.

Wound healing was registered by measuring the wound area not covered by epithelium in standardized, digital photographs. The size of the uncovered wound area was measured using planimetric software, a method reported in a review by Khoo and Jansen (2016), as having better precision and reliability than comparable methods. The reliability of measuring wound area in digital planimetry has been evaluated mainly in measuring chronic wounds and has been found to have high intra‐examiner reliability and acceptable inter‐examiner reliability (Stacy, Phillips, Farokhyar, & Swaine, 2017). However, it is necessary to photograph the wound with a marker of known dimension, usually a ruler, to enable calibration of linear dimensions. In this study, the edge of tooth 11 was measured in all patients and accordingly used as a marker for linear dimensions. Using a permanent structure located in the mouth, instead of holding a ruler in the mouth, simplifies the process of photographing children. Foltynski et al. (2015) have shown that the precision and accuracy of measurements further increases when two markers are placed above and below the wound. Thus, the precision, and validity, of this study may have been enhanced if both a tooth and a ruler had been used as markers. Intra‐examiner reliability was checked by measuring the uncovered wound surface of ten patients on standardized photographs immediately after, and 5 days after, surgery with three weeks interval after the first measurement. The test of intra‐examiner reliability showed a strong level of agreement. Pie‐Sanchez et al. (2012) describe how wound epithelization was complete 21 days after surgery using a CO_2_ laser, and 14 days after using an Er, Cr:YSGG laser, but the authors do not describe how this was measured. In this study the wound area not covered by epithelium was clearly larger immediately after laser surgery than after conventional surgery, but after 5 days we could not see any statistical differences between the groups in the size of wound surface. After 12 days all wounds in both groups were covered by epithelium.

Our initial hypothesis was that the wound would heal faster in the laser group and the power calculation was based on the wound healing 3 days faster in the laser group. However, at the 5‐day evaluations we could not see any statistical differences regarding wound surface not covered by epithelium. It is possible that we could have shown this difference if we had evaluated the wound surfaces every day between days 5 and 12, but this was not practically possible.

Delli et al. (2013) pointed out that, although there is a clinical interest in removing the frenum with laser surgery, there is no support for the view that this method is better than conventional methods. This study shows that frenectomy with Er:YAG laser is faster and causes less bleeding, but that there is no differences between the methods as regard to wound healing, reduction of midline diastema or patient's postoperative discomfort. As differences in treatment time and bleeding are of great clinical importance and the significantly shorter laser process may be crucial for some children being able to cope with the treatment, we consider that the Er:YAG laser frenectomy is preferable to conventional scalpel frenectomy, despite the fact that patients in this study value the methods equally.

**WHY THIS PAPER IS IMPORTANT TO PEDIATRIC DENTISTS**

- Evaluation of new methods with randomized controlled trials is important for the development of treatment methods in paediatric dentistry.
- This study evaluates the Er:YAG laser method in frenectomy and shows that it is faster, causes less bleeding and should be preferred to conventional scalpel technique in paediatric dentistry.

## Data Availability

The data that support the findings of this study are available from the corresponding author upon reasonable request.
